# First-Principles Calculations of Magnetite (Fe_3_O_4_) above the Verwey Temperature by Using Self-Consistent DFT + *U* + *V*

**DOI:** 10.1021/acs.jctc.3c00860

**Published:** 2023-11-17

**Authors:** Nelson Naveas, Ruth Pulido, Carlo Marini, Pierluigi Gargiani, Jacobo Hernandez-Montelongo, Ivan Brito, Miguel Manso-Silván

**Affiliations:** † Departamento de Física Aplicada, Universidad Autónoma de Madrid, 28049 Madrid, Spain; ‡ Departamento de Ingeniería Química y Procesos de Minerales, Universidad de Antofagasta, Avenida Angamos 601, 1270300 Antofagasta, Chile; § Instituto Universitario de Ciencia de Materiales “Nicolás Cabrera” (INC), Universidad Autónoma de Madrid, Campus de Cantoblanco, 28049 Madrid, Spain; ∥ Departamento de Química, Universidad de Antofagasta, Avenida Angamos 601, 1270300 Antofagasta, Chile; ⊥ CELLS−ALBA Synchrotron, 08290 Cerdanyola del Valles, Spain; # Departamento de Ciencias Matemáticas y Físicas, UC Temuco, 4813302 Temuco, Chile; ¶ Centro de Microanálisis de Materiales, Universidad Autónoma de Madrid, Campus de Cantoblanco, 28049 Madrid, Spain

## Abstract

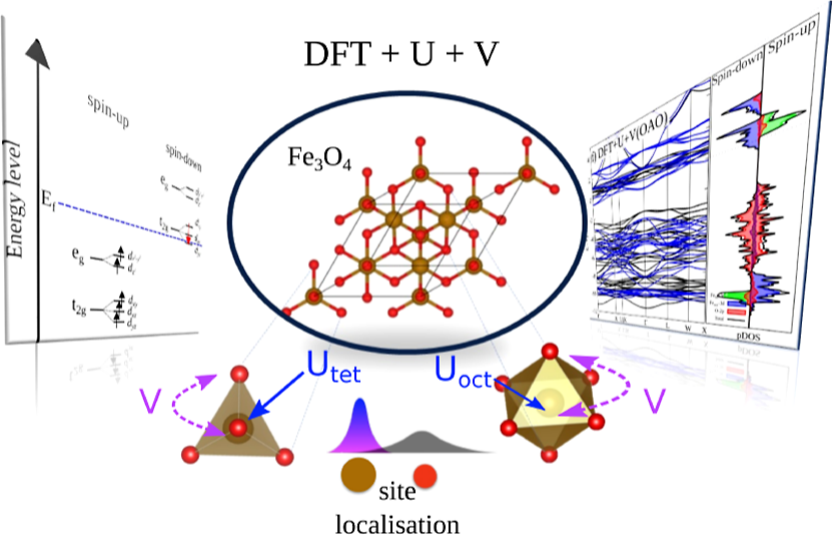

In this report, we have used the DFT + *U* + *V* approach, an extension of the DFT + *U* approach that takes into account both on-site and intersite interactions, to simulate structural, magnetic, and electronic properties together with the Fe and O K-edge XAS spectra of Fe_3_O_4_ above the Verwey temperature (*T*_v_). Moreover, we compared the simulated XAS spectra with experimental XAS data. We examined both orthogonalized and nonorthogonalized atomic orbital projectors and compared DFT + *U* + *V* to DFT, DFT + *U*, and HSE as a hybrid functional. It is noteworthy that, despite the widespread use of the same Hubbard *U* value for Fe_oct_ and Fe_tet_ at the DFT + *U* level in the literature, the HP code identified two distinct values for them using the Hubbard approaches (DFT + *U* and DFT + *U* + *V*). The resulting Hubbard *U* and *V* parameters are strongly dependent on the chosen orbital projectors. This study demonstrates how DFT + *U* + *V* can improve the structural, magnetic, and electronic properties of Fe_3_O_4_ compared to approximate DFT and DFT + *U*. In this context, DFT + *U* + *V* supports the half-metallic character of the bulk crystal Fe_3_O_4_ above *T*_v_, since the Fermi level is found in the t_2g_ band with a Fe_oct_ down-spin. Thus, the observations in the current study emphasize the significance of intersite interactions in the theoretical analysis of Fe_3_O_4_ above the *T*_v_.

## 1 Introduction

Despite being the preferred method for describing the electronic structure of condensed matter in materials science and engineering,^1−3^ approximate density functional theory (DFT) faces significant difficulties in adequately modeling the electronic structure and magnetic properties of transition-metal oxides, such as iron oxides.^4^ In part, this is due to the confined character of the Fe 3d orbitals and the substantial self-interaction error (SIE) of commonly used exchange–correlation functionals, including local density approximation (LDA) or generalized gradient approximation (GGA).^5−7^ To address SIE issues, a large number of studies employ hybrid DFT or DFT + *U*. On the one hand, hybrid DFT combines elements of both Hartree–Fock (HF) theory and approximate DFT functionals. Unfortunately, hybrid functionals are incredibly expensive compared to approximate DFT.^8,9^ On the other hand, in the DFT + *U* approach,^10,11^ an additional term is introduced into the approximate DFT energy functional, which describes the on-site Coulomb repulsion between electrons in localized d or f orbitals, while the remaining valence electrons are treated by the approximate DFT functionals. This term is derived from the simplified Hubbard model for strongly correlated electron systems and is typically denoted as *U*.^12^ Although DFT + *U* combines increased accuracy with a low computational cost, it only considers on-site interactions. As a result, it cannot adequately represent electronic hybridization in some materials because intersite interactions between an atom and its ligands are ignored. As a solution, the DFT + *U* + *V* method has been proposed as an extension, where *V* stands for intersite interactions. Thus, the DFT + *U* + *V* approach employs electronic interactions at both the on-site *U* and intersite *V* levels, adequately characterizing the intense intersite electronic hybridization in compounds made of transition metals.^13^ In the DFT + *U* + *V* method, a corrective term is added to the approximate DFT energy functional to improve the accuracy with which strongly correlated systems are described^13^
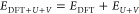
1

Here, the total energy of the system is composed of the *E*_DFT_, which represents the approximate DFT energy, while *E*_*U*+*V*_ symbolizes the Hubbard term, that either incorporates the on-site *U* and intersite *V* Hubbard parameters. As in the simple DFT + *U* case, in DFT + *U* + *V*, the Hubbard *U* parameter represents the effective strength of the on-site Coulomb repulsion while the additional Hubbard parameter *V* explains how electrons interact effectively with neighboring sites, a crucial aspect for compounds that require orbital hybridization among different atoms. Inside the simple rotationally invariant model, the extended Hubbard term is expressed as follows
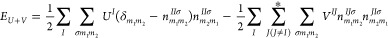
2

The atomic occupation matrices are determined by a generalized projection of the KS states onto the localized orbitals of neighboring atoms
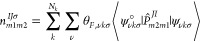
3

Thus, both DFT + *U* and DFT + *U* + *V* are Hubbard-corrected DFT methods that improve accuracy while using less computing power than, for example, hybrid functionals. However, when these Hubbard-corrected DFT methods are used, the values of the Hubbard parameters are not known in advance. Calculating the Hubbard parameters from first-principles can be challenging as they are typically determined empirically or by fitting experimental data.^14−17^ However, there are several computational methods designed to calculate Hubbard parameters, such as the constrained DFT (cDFT) approach,^18−25^ Hartree–Fock-based approaches,^26−31^ the constrained random phase approximation (cRPA),^32−37^ a linear-response formulation of cDFT, and even a machine learning-based approach,^16^ among others. Nevertheless, there are some problems with these methods, such as the need to use supercells and expensive computing power. In order to overcome these issues, by using the density-functional perturbation theory (DFPT) framework, Timrov has recently updated the Hubbard *U* linear response calculation.^38^ Instead of using finite differences across supercell calculations, this method uses summing over monochromatic (wave-vector-specific) perturbations in primitive cells, making it more computationally efficient than the prior method. Moreover, the numerical stability and convergence are improved, and the computing process is simplified via enhanced automation. Also, via this formulation, it is possible to compute the intersite *V* parameters.^39^

In this report, we used DFT + *U* + *V* to investigate magnetite (Fe_3_O_4_) above the Verwey temperature (*T*_v_) and compared it to approximate DFT, DFT + *U*, along with a hybrid functional (HSE). Magnetite is an attractive and complex magnetic material with unique electronic and magnetic properties, making it a topic of intense interest in basic and applied research. It exhibits a notable phase transition at a specific temperature called the Verwey temperature (near *T*_v_ ≈ 120 K), accompanied by a first-order metal–insulator behavior transition.^40^ Although numerous aspects of the Verwey transition in bulk magnetite crystals have been researched since Verwey’s seminal paper in 1939^41^ (especially below *T*_v_), there is still an intense debate considering if this material presents a semiconductor or half-metallic nature above the *T*_v_. In fact, it has been an important question whether the disordered charge, orbital, and trimeron correlations persist in the high-temperature cubic phase.^42^ In the present paper, we hypothesized that applying the DFT + *U* + *V* theory to investigate magnetite above the Verwey temperature transition can lead to a more accurate understanding of the material properties by considering both the on-site and intersite electronic correlation effects that are significant in this material. Our research focuses on the room-temperature cubic phase as this is where the semiconductor or semimetallic debate arises as well as being relevant to environmental and biomedical applications. Therefore, we first computed the on-site *U* and intersite *V* Hubbard parameters self-consistently from first principles using density functional perturbation theory (DFPT). Second, the Fe_3_O_4_ structure was optimized using the converged *U* and *V* Hubbard parameters. Finally, we examine the structural, magnetic, and electronic characteristics of Fe_3_O_4_ above the Verwey temperature transition using DFT + *U* + *V*.

## 2 Methods

### 2.1 DFT Calculation Details

**Figure 1:**
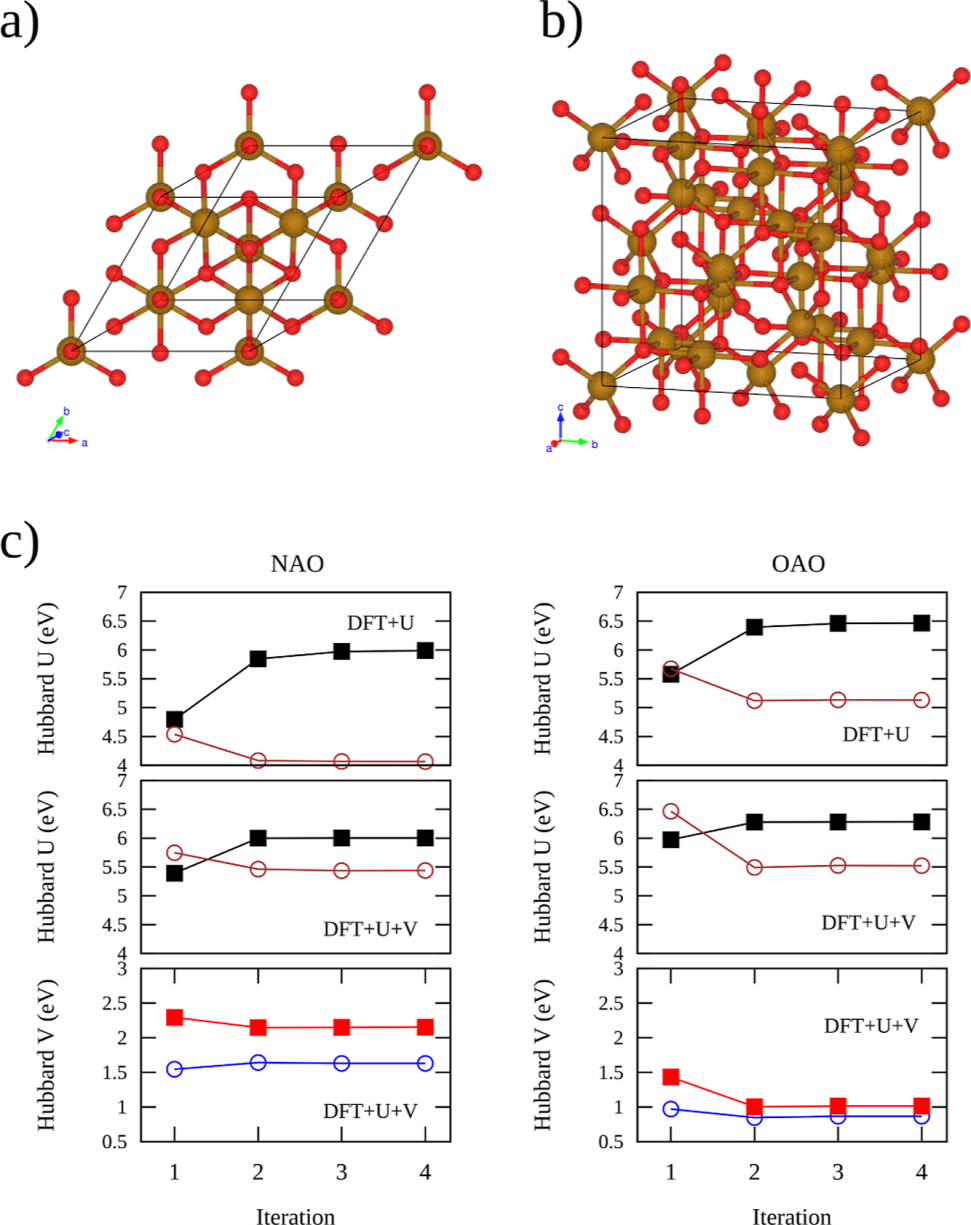
Crystal model of the (a) primitive and (b) unit cell of the Fe_3_O_4_. (c) Progression of Hubbard parameters (*U* and *V*) for bulk Fe_3_O_4_ behind each iteration of the self-consistent calculations for both DFT + *U* and DFT + *U* + *V*. Calculations were performed by using either NAO or OAO projectors. In the Hubbard *U* curves, the black and brown lines represent the Fe_oct_ and Fe_tet_ Hubbard *U* values, respectively. Similarly, the Hubbard *V* parameters associated with the shorter and longer Fe–O distances are represented by the red and blue lines in the Hubbard *V* curves.

DFT calculations were computed with the Quantum Espresso computational package^43^ using the plane-wave (PW) pseudopotential approach. In our study, we used both PBEsol and PBE pseudopotentials downloaded from the SSSP Precision Library v1.2.^44^ In all calculations, the ferrimagnetic properties of Fe_3_O_4_ and the spin polarization were taken into account because of the strong antiferromagnetic interaction between the tetrahedral (Fe_tet_) and octahedral (Fe_oct_) Fe ions. Then, the spins of Fe_oct_ and Fe_tet_ were set up and down, respectively. The rhombohedral primitive cell (Figure 1a), which has 14 atoms, and the conventional unit cell (Figure 1b), which has 56 atoms, were both employed.

For the calculation of the Hubbard parameters, we used the HP code contained in Quantum Espresso.^38,39,45^ The HP code utilizes DFPT to calculate Hubbard parameters (on-site *U* and intersite *V*) within the context of DFT + *U* and DFT + *U* + *V*.^45^ In DFPT calculations, we used the primitive cell with a uniform Γ-centered *k* and *q* point mesh of 5 × 5 × 5 and 2 × 2 × 2 *q* point mesh, along with kinetic-energy cutoffs of 65 and 780 Ry for the wave function and the charge density, respectively. At this point, the self-consistent approach to compute *U* and *V* parameters was used, involving cyclic computations of the optimized structures and the subsequent recalculation of Hubbard parameters for each newly optimized crystal structure.^39^

The BFGS quasi-Newton algorithm was used to optimize the crystal structures using the approximate DFT, DFT + *U*, and the extended DFT + *U* + *V* approaches. For total energy, forces, and pressure, we set convergence thresholds of 1 × 10^–6^ Ry, 1 × 10^–5^ Ry/Bohr, and 0.5 kbar, respectively. By applying a Hubbard *U* correction to Fe 3d states, DFT + *U* computations have been performed employing the simplified rotational-invariant formulation used by Cococcioni and de Gironcoli.^46^ Also, utilizing the simplified version of Leiria Campo and Cococcioni,^13^ the intersite interaction among the Fe 3d and O 2p orbitals was corrected employing the Hubbard *V* parameter in DFT + *U* + *V* calculations. Nonorthogonalized atomic orbitals (NAOs) and orthogonalized atomic orbitals (OAOs) have been applied for the DFT + *U* and DFT + *U* + *V* computations.^39^ We used the Marzari–Vanderbilt (MV) smearing technique^47^ with a 1 × 10^–2^ Ry broadening parameter.

A uniform Γ-centered *k* point mesh of 10 × 10 × 10 was used to calculate structural optimizations, band gaps, magnetic moments, and the projected and total density of states (DOS). At this level, kinetic-energy cutoffs of 90 and 1080 Ry were used for the wave function and the charge density, respectively. The magnetic moment per site for Fe atoms, which we outline in our paper, is obtained from the SCF calculations. This moment is equivalent to a weighted integration of the electronic moment within a sphere surrounding the nucleus. To determine the charge of Fe and O ions in Fe_3_O_4_, Bader charge analysis was performed.^48−51^

### 2.2 Experimental and Simulated XAFS Details

We conducted X-ray absorption spectroscopy experiments while maintaining a vacuum environment at the CLÆSS beamline of the ALBA CELLS synchrotron in Spain. The K-edge of Fe (7124 eV) measurements were carried out in a continuous mode utilizing a double-crystal Si(311) monochromator. To ensure the accuracy of measurements, we checked the energy calibration of each collected spectrum by aligning the first derivative maximum (7112 eV) of the XAS reference spectrum of a Fe foil, which was acquired alongside the sample. The absorption data have been collected in the transmission mode utilizing three adequately gas-filled ionization chambers at this energy. All spectra within the XANES region were sampled with an energy resolution of 0.3 eV. The specifications have been handled in accordance with the standard procedure. Pre-edge background subtraction and XANES normalization were conducted using a linear polynomial fit for the pre-edge region and a cubic polynomial fit for the postedge region of the absorption spectrum. Spectra were also separated based on the absorption jump. The processing of data was conducted utilizing ATHENA software, which is included in the IFEFFIT suite.

XAS measurements in the soft X-rays have been performed at beamlines BL29-BOREAS at the ALBA synchrotron (Spain). The synchrotron radiation emitted by a pure permanent magnet helical undulator (capable of delivering both linearly and circularly/elliptically polarized light) is first intercepted by a plane and toroidal mirrors and then by the monochromator. This latter one is based on variable line spacing plane gratings, working at a fixed included angle of 175 or 177° in combination with dedicated spherical mirrors. Finally, two bendable mirrors arranged in Kirkpatrick–Baez geometry focus the beam on the sample. At the K-edge (543 eV), polarized XANES spectra were measured in total electron yield on sintered pieces. The incident X-ray flux measured as the TEY current on a clean gold mesh between the last optical element and the sample normalized the sample signal. All of the XANES spectra presented have been normalized. Initially, the linear pre-edge contribution is subtracted and then the jump is fixed to 1 at values well above the absorption edge. There has been no normalization or other unmentioned data manipulation.

By employing the Lanczos recursive procedure inside of the XSpectra code of Quantum Espresso distribution,^52−54^ simulations of Fe and O K-edge XANES spectra were carried out. In our calculations, the dipolar part of Fe and O K-edge XANES spectra was considered, neglecting both the quadrupole part and the core-hole in the final state. The convolution of the XANES spectra was produced employing a Lorentzian smearing arctangentlike function with an energy-dependent broadening.^55^

The EXAFS signal of Fe_3_O_4_ has been extracted using the standard procedure previously reported.^56^ Briefly, corresponding Fourier transforms have been conducted over the *k* range 2.6–14 Å^–1^ once it has been multiplied by *k*^2^ in order to strengthen the signal in the higher *k*-region. The EXAFS oscillations have been modeled by utilizing the structures achieved through relaxation within DFT, DFT + *U*, and DFT + *U* + *V*. The FEFF-6 code^57^ was used to calculate the theoretical amplitudes and phases of the various paths used in the analysis. The ARTEMIS program of the DEMETER suite^58^ was then used to adapt the theoretical values to the experimental data (in *R* space).

## 3 Results and Discussion

### 3.1 Calculations of Hubbard Parameters

In the Fe_3_O_4_, the on-site *U* correction is essential for describing Fe 3d states and the intersite *V* parameter is anticipated to be effective for characterizing Fe 3d–O 2p hybridization. Hence, we have determined the Hubbard parameters (*U* and *V*) for Fe_3_O_4_ through the self-consistent calculation method proposed by Timrov et al., 2021.^39^Figure 1c shows the progression of the *U* and *V* Hubbard parameters for the Fe_3_O_4_, employing NAO or OAO orbital projectors after each iteration. Interestingly, despite the fact that the literature widely uses the same Hubbard *U* value for Fe_oct_ and Fe_tet_, here the HP code identified, by considering the occupation matrix, two different values for them. Regardless of the Hubbard-corrected theory utilized, the progression of *U* values, illustrated in Figure 1c, displays divergent trends for both Fe_oct_ and Fe_tet_. The values for Fe_oct_ increased, while those for Fe_tet_ decreased from the initial to the final iteration. In general, the self-consistent procedure results in higher converged *U* values for Fe_oct_ than Fe_tet_ for both DFT + *U* and DFT + *U* + *V*. On the one hand, when DFT + *U* corrections are applied, the converged values of *U* depend strongly on the projector used. We find that the Hubbard *U* values for both Fe_oct_ and Fe_tet_ are consistently higher when using the OAO projectors compared to the NAO projectors. On the other hand, if the corrections are a DFT + *U* + *V* level, the dependence of the *U* values on the projector used is not as strong as with DFT + *U* (the difference between NAO and OAO values is reduced). On the contrary, the projector used has a significant effect on the converged values of *V*, resulting in higher values when using NAO projectors than OAO. Similarly to the determined *U* values obtained, here the HP code identified two different values for the *V* parameter, one corresponding to Fe_oct_–O and the other to Fe_tet_–O interaction.

In a previous study, linear response calculations using DFT + *U* theory revealed different self-consistent *U* values for Fe_oct_ (4.7 eV) and Fe_tet_ (4.3 eV).^59^ These results are consistent with our research, where we detected distinct *U* values for both Fe_oct_ and Fe_tet_, with Fe_oct_ showing a *U* value higher than Fe_tet_. Nevertheless, in our study, we found a greater contrast between Fe_oct_ and Fe_tet_*U* values using DFPT. Differences in pseudopotential types and exchange–correlation functionals employed can account for the divergences between our outcomes and those of prior research.^45,60,61^ Additionally, our research emphasizes the significance of precisely determining the Hubbard *U* parameter and orbital projector selection, as well as the consideration of intersite interactions through the application of DFT + *U* + *V* theory.

The structural optimization involved in the self-consistent technique is essential to achieving the final Hubbard parameter values in Fe_3_O_4_ as previously described for hematite (α-Fe_2_O_3_),^62^ another widely used iron oxide. The importance of this process has also been demonstrated for other types of materials, such as SrTiO_3_,^63^ MnPO_4_, LiMnPO_4_,^39^ and Fe-doped α-MnO_2_.^64^ The obtained Hubbard parameters after the fourth iteration are summarized in Table 1. The Hubbard parameters are sufficiently converged for the majority of postprocessing applications when they are within 0.01 eV for the DFT + *U* and DFT + *U* + *V* approaches after the subsequent iterations.

**Table 1:** Convergence Evolution of the Hubbard Parameters, *U* and *V*, Computed through DFPT for the Bulk Crystal Cell of Fe_3_O_4_

method	manifold	Hubbard *U* (eV)	Hubbard *V* (eV)
		Fe_oct_/Fe_tet_	Fe_oct_–O/Fe_tet_–O
DFT + *U*	NAO	5.9877/4.0631	
DFT + *U* + *V*	NAO	6.0008/5.44	1.6295/2.1545
DFT + *U*	OAO	6.4674/5.1323	
DFT + U + V	OAO	6.2825/5.5229	0.8661/1.0145

### 3.2 Calculation of Structural Parameters

After determining the Hubbard parameters, we compared the experimental lattice parameter (*a*) and the residual volume (*V*) of the conventional Fe_3_O_4_ unit cell with those predicted by the different theories applied in our study. Note that Fe_3_O_4_ has a cubic inverse spinel structure (space group *Fd*3*m*) at room temperature, and there are a number of eight formula units in its conventional unit cell, which include 24 Fe and 32 O atoms. In our investigation, the crystal structure of Fe_3_O_4_ was fully relaxed at the different studied theories (DFT, DFT + *U*, and DFT + *U* + *V*), preserving its cubic symmetry. Table 2 compares the experimental value with the optimized lattice parameter (*a*) and the volume (*V*) of the Fe_3_O_4_ unit cell as achieved by different theories. The standard DFT approach, as previously reported for Fe_3_O_4_, subestimates the lattice parameter (1.47%) and the residual volume (4.35%). Although the Hubbard-corrected DFT approaches (the simple DFT + *U* and the extended DFT + *U* + *V*) result in different tendencies, they enhance the match against the volume and the experimental lattice parameter *a*. According to the Hubbard-corrected method that was utilized, the discrepancy for the calculated and experimental values drops to a range of 0.8–1.05% for DFT + *U* and 0.6–0.7% for DFT + *U* + *V*. In fact, it has been extensively reported that the DFT + *U* method (containing just the on-site correction) has improved the description of the structural properties of Fe_3_O_4_ and other transition-metal oxides. However, the DFT + *U* + *V* approach has not received the same level of attention. Also noteworthy is the fact that the Hubbard projector type significantly affects the resultant lattice parameter achieved by DFT + *U* and DFT + *U* + *V*. For example, we observe that DFT + *U* overestimates the lattice parameters by 1.05% when NAO projectors are used. On the other hand, the lattice parameter by DFT + *U* + *V* is subestimated by just 0.74%. Therefore, the best match with experiments was then discovered for NAO projectors to be at the DFT + *U* + *V* level.

**Table 2:** Structural Parameters of the Unit Cell of Bulk Fe_3_O_4_ Computed via DFT, DFT + *U*, and DFT + *U* + *V* Theories

Hubbard manifold	method	*a* (Å)	*V* (Å)
	Exp	8.396	591.858
	DFT	8.272	566.092
NAO	DFT + *U*	8.484	610.768
NAO	DFT + *U* + V	8.333	578.744
OAO	DFT + U	8.463	606.095
OAO	DFT + U + V	8.446	602.568

**Figure 2:**
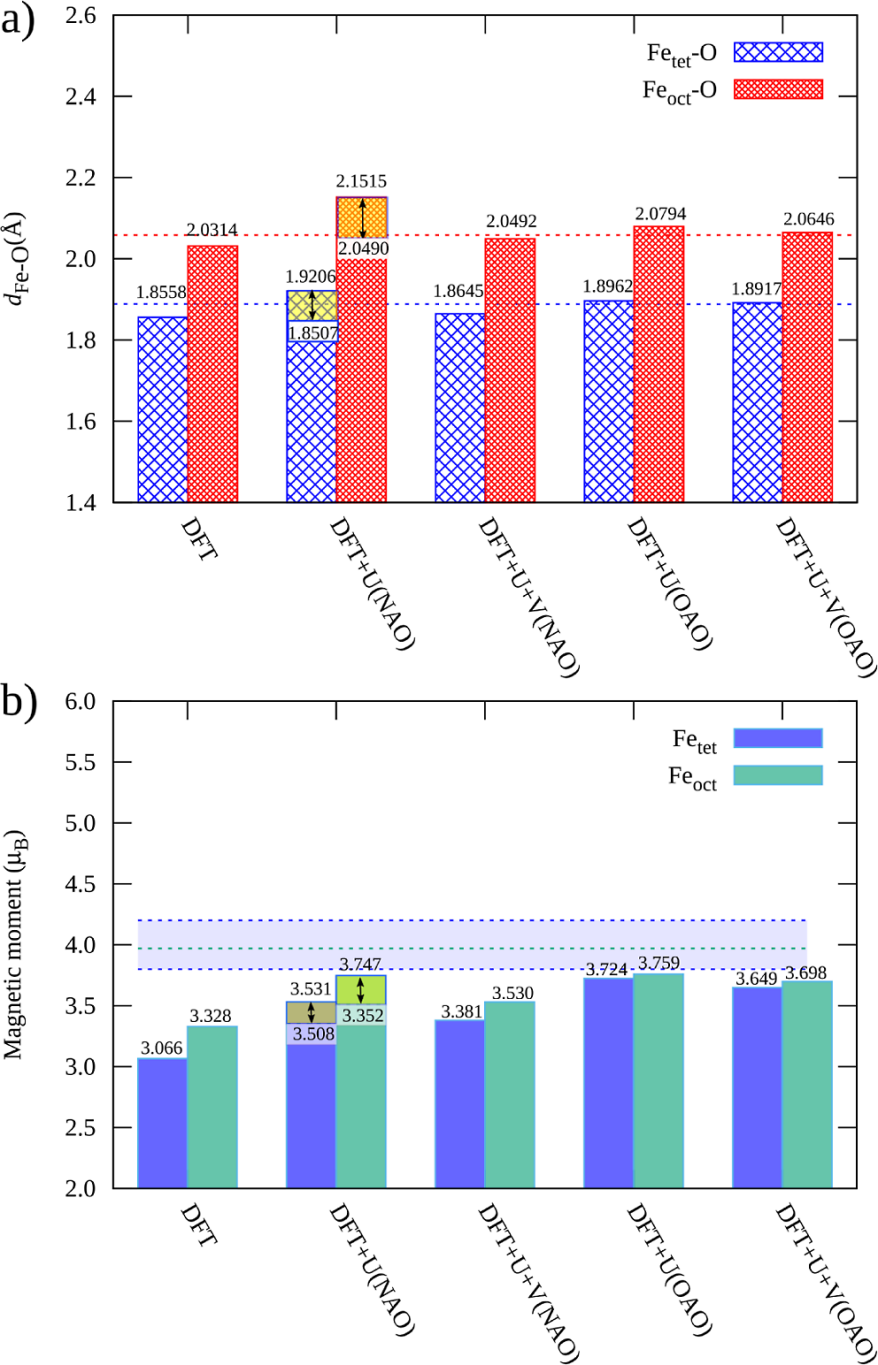
(a) Optimized Fe–O bond lengths and (b) Fe magnetic moment in the crystal cell of Fe_3_O_4_ calculated via DFT, DFT + *U*, and DFT + *U* + *V* theories. Dash lines correspond to the experimental data of Fe–O bonds and magnetic moments as appropriate.

Figure 2a shows the optimized Fe–O bond lengths in the conventional unit cell of Fe_3_O_4_ computed by DFT, DFT + *U*, and DFT + *U* + *V* theories. Experimentally, two Fe–O bonds can be distinguished by looking at the bond lengths in the conventional unit cell by using neutron powder high-resolution diffraction,^65^ one of which is shorter and measures around 1.8885 Å, while the other is longer and measures around 2.0582 Å. These two Fe–O bonds correspond to those formed by Fe_tet_ and Fe_oct_, respectively. As occurred for the lattice parameter results, approximate DFT underestimates both Fe–O bonds. In this sense, all Hubbard-corrected theories improve the results of both Fe–O bonds. However, the simpler of the Hubbard-corrected theories, DFT + *U*-NAO, shows a different pattern for the Fe–O bond lengths. This theory results in several distances for Fe_tet_–O and Fe_oct_–O due to the loss of crystalline symmetry after the structural optimization process. Interestingly, the rest of the Hubbard-corrected theories show just two Fe–O bond lengths, where DFT + *U* + *V* better describes their distances.

### 3.3 Magnetic Properties and Charge Analysis

Fe_3_O_4_ has a magnetic moment of +4 μ_B_ per formula unit in the basic ferrimagnetic model, which is completely attributed to the Fe_oct_^2+^ cations. The Fe_tet_^3+^ and Fe_oct_^3+^ cations cancel one another out.^66^ This supports the hypothesis of half-metallic conduction and compares favorably with the experimental measurement of 4.07 μ_B_.^67^Figure 2b shows the magnetic moments on Fe atoms in Fe_3_O_4_ calculated by the different investigated theories. The experimental magnetic moment values for the Fe_oct_ and Fe_tet_ are 3.97^65^ and 3.8–4.2 μ_B_,^65,68^ respectively, as shown by dashed lines in Figure 2b. The magnetic moments calculated by DFT are 3.328 and −3.066 μ_B_ for Fe_oct_ and Fe_tet_, respectively, which are drastically smaller than the experimental values. Interestingly, the Hubbard corrections improve the estimation in all situations, although the results depend on the used theory as well as the used orbital projector. Additionally, they still underestimate the magnetic moment by 0.4–0.8 μ_B_. Similar to the Fe–O distance result, DFT + *U*-NAO results in several magnetic moments for Fe_oct_ and Fe_tet_ because, under this theory, structural optimization causes a loss of symmetry. At the DFT + *U* level, the calculated magnetic moments for the Fe_oct_ and Fe_tet_ were 3.6792 and −3.5773 μ_B_ for NAO and 3.759 and −3.724 μ_B_ for the OAO projectors. When using DFT + *U* + *V* theory, the calculated magnetic moments for the Fe_oct_ and Fe_tet_ were 3.530 and −3.381 μ_B_ for NAO and 3.698 and −3.649 μ_B_ for the OAO projectors. Thus, the better agreement with the experimental results is DFT + *U* and DFT + *U* + *V* when using OAO projectors.

In order to determine the influence of the Hubbard correction theories on the Fe partial charges for the Fe_3_O_4_, we performed a Bader charge analysis (Table S1). On the one hand, similarly to the Fe–O distances and magnetic moment results, DFT + *U*-NAO yields multiple charges for Fe_oct_ (3.438 e/3.621 e) and Fe_tet_ (3.654 e/3.681 e). On the other hand, at the DFT + *U*-NAO, DFT + *U* + *V*-NAO, and DFT + *U* + *V*-OAO levels, the charge coming from the Fe_tet_ and Fe_oct_ can be distinguished (Table S1). In addition to the Bader analysis, we have also carried out the method proposed by Sit et al. (2011)^69^ to determine the oxidation states (OS) of Fe. This procedure involves separating the contributions of orbital mixing to the assigned charge from those due to the actual occupation of the d-orbitals. However, the application of this procedure to our system was challenging due to the presence of multiple Fe sites. In fact, only DFT and U-NAO give multiple Fe OS values for Fe_oct_ (Fe^2+^ and Fe^3+^). Nonetheless, of the 16 Fe_oct_ sites, only 4 are Fe^2+^, indicating incomplete charge disproportionation. The remaining theories are unable to discriminate among Fe_oct_ of different OS, leading to an OS of Fe^3+^ for all sites (see Table S2).

### 3.4 Electronic Properties

The electronic structure of Fe_3_O_4_ has been studied using a wide variety of experimental approaches, such as angle-resolved photoelectron spectroscopy using synchrotron radiation,^70^ optical-conductivity spectra,^71^ and photoemission spectroscopy,^72,73^ among others.

**Figure 3:**
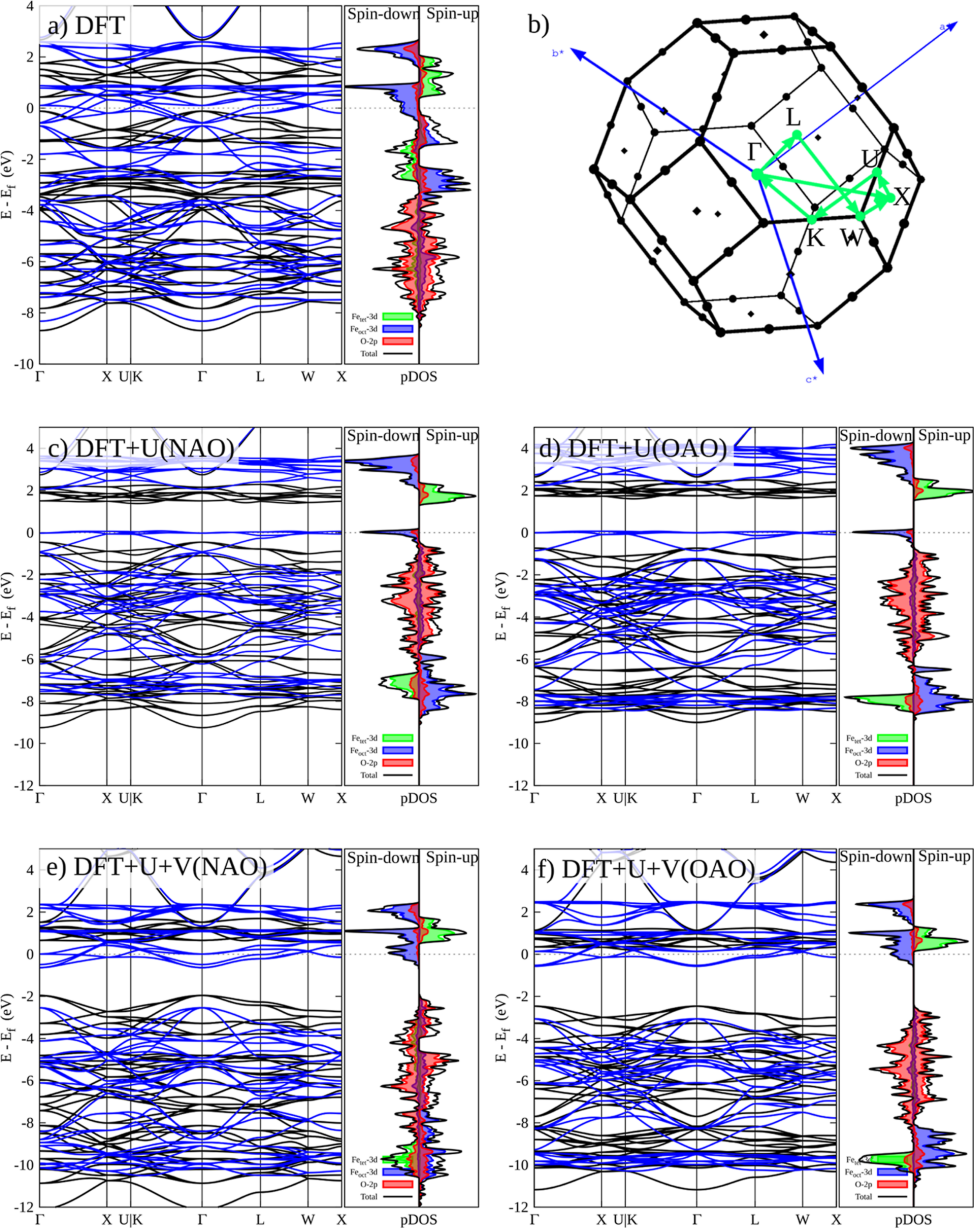
Band structures and pDOS for the Fe_3_O_4_ bulk crystal calculated by DFT (a), DFT + *U* (c,d for NAO and OAO, respectively), and DFT + *U* + *V* (e,f for NAO and OAO, respectively). The selected path in the reciprocal space is shown in (b).

Figure 3 depicts the band structure and partial density of states (pDOS) of the Fe_3_O_4_ bulk crystal calculated through conventional DFT and different corrected Hubbard approaches by using NAO and OAO projectors. The band structure and the pDOS projected on the Fe_oct_, Fe_tet_, and O sites obtained by the DFT (Figure 3a) show that the Fe_3_O_4_ is a half-metallic oxide with a spin-up channel that displays semiconducting behavior and a spin-down channel that exhibits metallic behavior, which is in accordance with previous reports using LSDA^74^ and PBE^75,76^ XC functionals. The spin-up band structure produced by the approximate DFT theory has an electronic structure with a direct band gap of 0.3 eV at the Γ point. In the vicinity of the Fermi energy, the pDOS indicates substantial hybridization of the O 2p and Fe 3d states. The conduction band is mostly made up of Fe 3d states, where the spin-up and down are composed of Fe_tet_ and Fe_oct_, respectively. This behavior is reversed in the valence band, where the spin-up is composed of Fe_oct_ and the spin-down of Fe_tet_. Likewise, according to the pDOS, the O 2p states are localized in the valence band region between −3.0 and −8.0 eV. As a consequence of the SIE related to the approximate DFT, Fe 3d states are overdelocalized owing to their significant hybridization with O 2p states.

Figure 3c–f shows the band structure and pDOS of Fe_3_O_4_ by using corrected Hubbard approaches. At the DFT + *U* level, the result of the band structure and pDOS does not depend significantly on the orbital projector used (Figure 3c,d). Indeed, there are only small differences in the intensities of Fe states between DFT + *U* with NAO and OAO. On the one hand, the semiconducting nature of the electron spin-up of Fe_3_O_4_ is accurately described by the DFT + *U* theory, which results in a greater band gap (1.6 eV for NAO and 2.1 eV for OAO) than the approximate DFT. On the other hand, there exists a finite DOS at the Fermi energy in the spin-down channel. This is in line with previous results, where the electronic structure of Fe_3_O_4_ is described as half-metallic by using DFT + *U* theory. For both NAO and OAO projectors, the minimum of the conduction band is dominated by Fe 3d states, with Fe_oct_ at the spin-down channel and Fe_tet_ at the spin-up channel. Although the O 2p states dominate at the maximum of the valence band, finite and localized Fe_oct_ states persist, indicating the hybridized states in this area. Also, most of the Fe 3d states, which were originally close to the Fermi level in approximate DFT, have shifted to higher binding energy due to Coulomb interactions between Fe 3d electrons. The main difference in this region between the NAO and OAO projectors is that the OAO projector evidenced a colocalization between Fe_oct_ (spin-down) and Fe_tet_ (spin-up) at low energies around −8.0 eV. Interestingly, in contrast to approximate DFT theory, under the application of DFT + *U*, the O 2p states are localized at higher energies in the valence band region between −0.5 and −5.8 eV. The inclusion of the Hubbard *V* parameter through DFT + *U* + *V* calculations induces significant modifications to both the electronic structure of Fe_3_O_4_ (Figure 3e,f) in comparison to DFT + *U*. Despite the differences, DFT + *U* + *V*, like DFT + *U*, also supports the half-metallic nature of bulk crystal Fe_3_O_4_ above *T*_v_, given that the Fermi level resides in a Fe_oct_ 3d down-spin state. A major difference between DFT + *U* + *V* and DFT + *U* is that, in DFT + *U* + *V*, the down-spin Fe_oct_ 3d located at the Fermi energy exhibits a broader energy range. Another important difference is that DFT + *U* + *V* results in a greater band gap (2.3 eV for NAO and 2.4 eV for OAO) than the approximate DFT + *U* for the electron spin-up channel. Thus, DFT + *U* + *V* gets a better description of the semiconducting nature of the majority electron spin of Fe_3_O_4_ with a bigger band gap than DFT + *U* and a metallic behavior of the minority spin.

**Figure 4:**
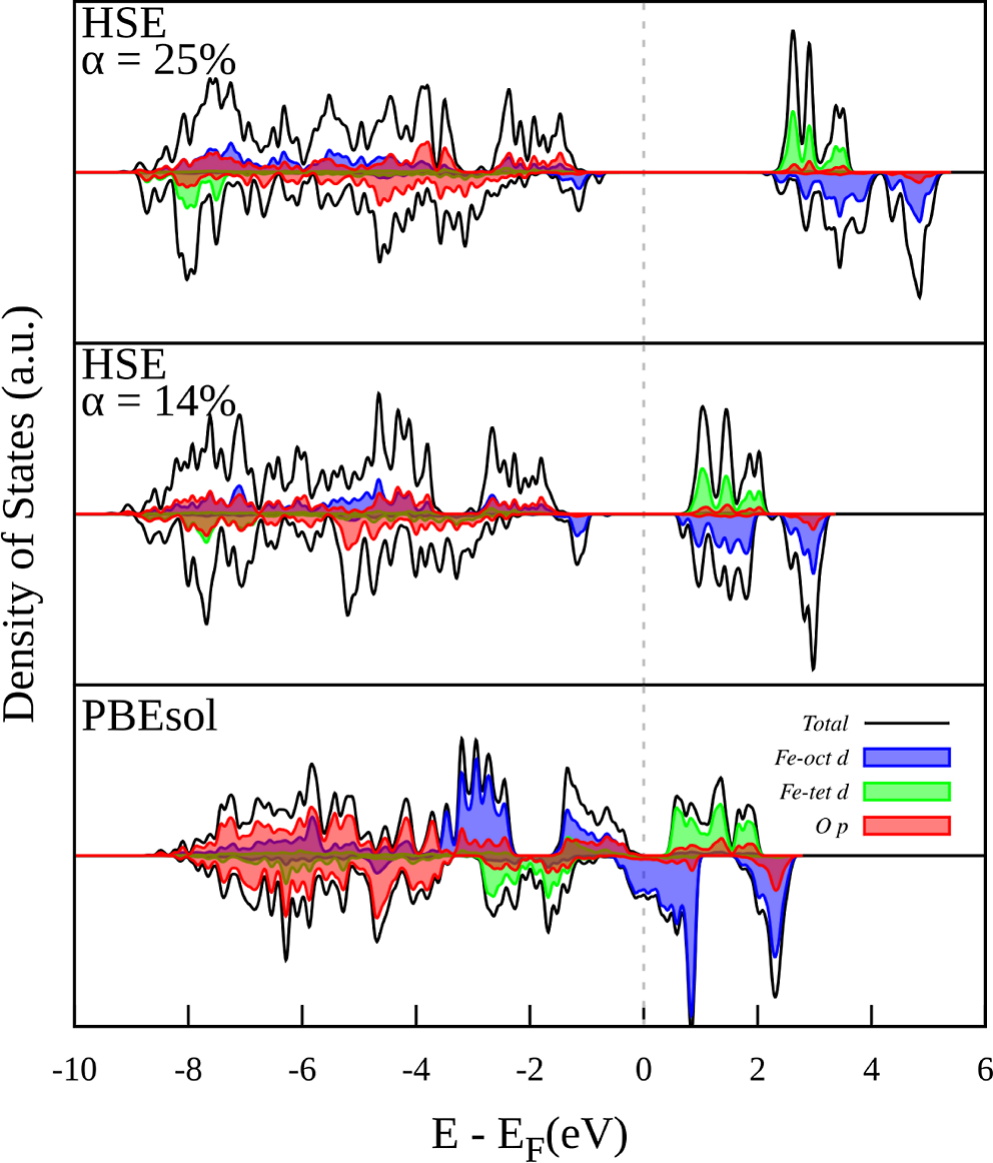
Comparison of the pDOS for the Fe_3_O_4_ bulk crystal calculated by DFT (PBEsol) and HSE hybrid functional at different exchange fractions (α = 14% and α = 25%).

Figure 4 compares the conventional DFT employed in this work (PBEsol) with the HSE hybrid DFT functional to exhibit the total DOS and pDOS estimated for Fe_3_O_4_. Here, to simulate the DOS and pDOS for the HSE functional, an SCF calculation was performed on the structure that had already been optimized using DFT + *U* + *V*-OAO. Different exchange fractions were used to demonstrate the necessity of refining this parameter in order to get a specific result. In fact, this indicates that using hybrid functionals is not a full first-principles method. Also, it is important to note that the utilization of hybrid functionals like HSE is considerably less efficient and more costly compared to approximate DFT and Hubbard-corrected DFT approaches. As shown in Figure 4, the HSE functional cannot describe the symmetrical charge distribution across Fe_oct_ atoms of the unit cell seen at room temperature, resulting in a semiconducting behavior for both spin channels. Due to charge order, which is also characterized by the distribution of magnetic moments among Fe_oct_ atoms and the emergence of a d–d optical band gap, symmetry breaking may lead to distortions of Fe_oct_.^77^ This result has been a topic of discussion for years.^75−77^ For example, Liu and Di Valentin, 2017^76^ have demonstrated that using no-symmetry restriction, DFT + *U* theory using a Hubbard *U* = 3.5 eV provokes opening a gap around the Fermi energy, thus causing a semiconducting behavior in the ground state of Fe_3_O_4_. Our results do not show a semiconducting behavior, probably due to the lack of disproportionation of Fe states. However, when applying no-symmetry, the only theory used in this study that opens a complete gap around Fermi energy is DFT + *U*-NAO. In fact, in this case, the asymmetric ground state has a lower total energy than the symmetric state (Δ*E* = −0.47 eV; Table S3). Neither DFT + *U*-OAO nor DFT + *U* + *V* theories open a gap when using the no-symmetry restriction (Figure S1). We have also performed calculations with DFT + *U*-OAO, DFT + *U* + *V*-NAO, and DFT + *U* + *V*-OAO theories starting from the symmetry-broken DFT + *U*-NAO structure to assess whether the charge order would arise spontaneously. However, in all cases, it is found that the symmetric ground state has a lower total energy than the asymmetric state (Δ*E*_+*U*NAO_ in Table S3).

**Figure 5:**
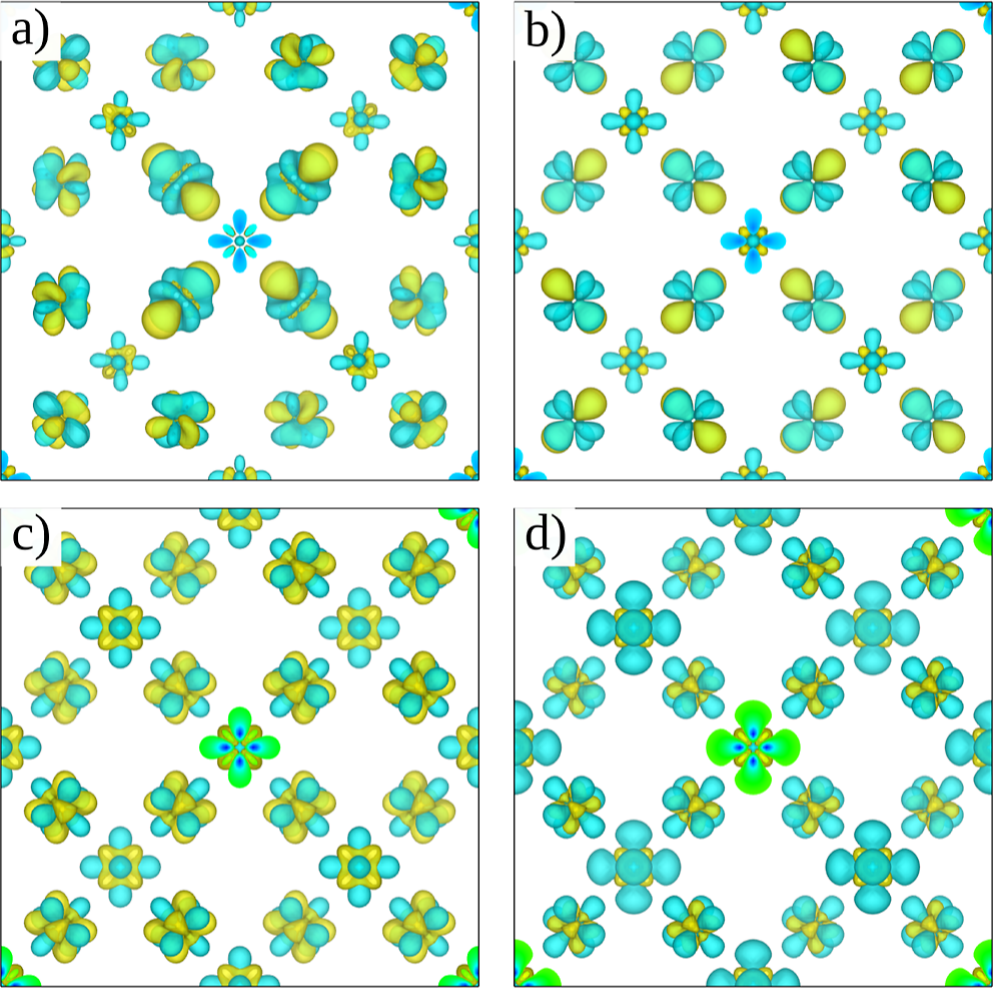
Electron density difference between Hubbard-corrected DFT and approximate DFT at a fixed lattice parameter. (a) DFT + *U* (NAO), (b) DFT + *U* (OAO), (c) DFT + *U* + *V* (NAO), and (d) DFT + *U* + *V* (OAO). Yellow and green colors represent the gain and loss of electron density, respectively.

Figure 5 shows the changes in electron density at a fixed lattice parameter by using the different Hubbard-corrected theories applied in this study. First, it is interesting to note that the loss and gain of electron density from the area directly surrounding the Fe center has a δ-orbital character for all the theories. This behavior has been reported previously for other 3d transition-metal oxides^78^ under DFT + *U* theory. In the case of DFT + *U* (Figure 5a,b), the loss of electron density from the region immediately around the Fe center and the increase of electron density in the regions surrounding the O atoms are observed. On the one hand, when using DFT + *U*-NAO, due to the loss of crystalline symmetry after the structural optimization process, it is possible to appreciate different topologies for the loss and gain of electron density around the Fe center. On the other hand, when using DFT + *U*-OAO, two topologies are clearly shown, which correspond to the electron density around the Fe_tet_ and Fe_oct_ center. In the case of DFT + *U* + *V*, it is also possible to clearly identify the electron density coming from Fe_tet_ and Fe_oct_, although some interesting changes in the topology of the electron density appear. Moreover, when comparing the used projectors inside the DFT + *U* + *V* theory, it can be seen that by using OAO projectors, the loss of electron density increases.

In order to comprehend the influence of the Hubbard parameters, *U* and *V*, we can observe the action of the Hubbard potential on Kohn–Sham wave functions by obtaining the functional derivative of *E*_DFT+*U*+*V*_ with respect to the complex conjugate of the same Kohn–Sham wave function^13,45^
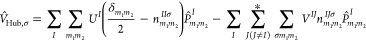
4

The two components in the corrective energy functional, which are proportional to the respective on-site (*U*^*I*^) and intersite (*V*^*IJ*^) couplings, work against one another. Although the on-site term favors localization on atomic sites (usually reducing hybridization), the intersite term favors hybridized states with components on neighboring atoms. In fact, when analyzing the occupation matrices of the Hubbard-corrected approaches in Tables S3–S6, it is possible to appreciate that the potential of DFT + *U* theory decreases the fractional occupations regardless of the projectors used. Interestingly, the effect of the potential of the DFT + *U* + *V* theory on fractional occupations strongly depends on the projector used. When using the DFT + *U* + *V*-NAO theory, the decrease in the fractional occupations is reversed almost completely, which is not the case for DFT + *U* + *V*-OAO. This differential behavior can be explained by the fact that the *V* Hubbard values in DFT + *U* + *V*-NAO are bigger than for DFT + *U* + *V*-OAO.

### 3.5 X-ray Absorption Fine Structure Spectroscopy

X-ray absorption fine structure (XAFS) spectroscopy has become an invaluable tool for studying the electronic structure of materials. The core concept behind XAFS is based on two different regions of the measured signal: X-ray absorption near edge structure (XANES) and extended X-ray absorption fine structure (EXAFS).^79^ The combination of these two types of data reveals key details about the chemical composition and overall structural properties of a sample. Then, the information derived from XAFS measurements can provide insights into bonding interactions, OS, coordination numbers, and other parameters related to the electronic properties of iron oxide materials.^80^

**Figure 6:**
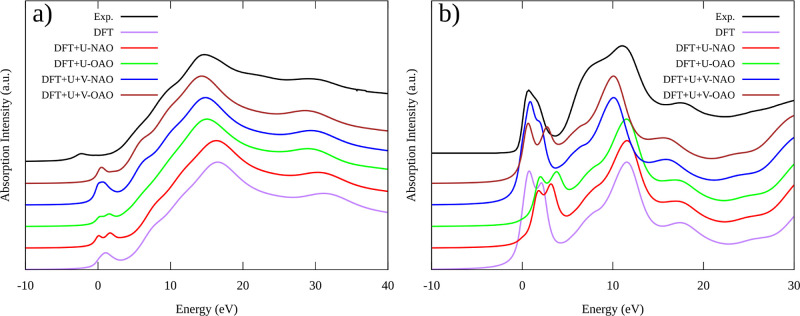
Simulation of Fe_3_O_4_ bulk (a) Fe and (b) O K-edge XANES through DFT, DFT + *U*, and DFT + *U* + *V* calculations using NAO and OAO projectors and comparison against respective experimental spectra (black lines).

In particular, XANES is a remarkably sensitive method for examining the valence state, ligand symmetry, coordination chemistry, and local structure close to the absorber atom. In fact, the study of magnetite has made considerable use of XANES spectroscopy over the years.^81−86^ In the XANES region, three distinctive features can be identified: the edge position (*E* = *E*_0_), the pre-edge region (*E* < *E*_0_), and the postedge region (*E* > *E*_0_). Thus, we are going to analyze our simulations according to these characteristic features. Figure 6 shows the experimental and calculated Fe and O K-edge XANES spectra of Fe_3_O_4_ by using both uncorrected and corrected Hubbard approaches. Additionally, it can be seen from Figure S2 that disregarding the core–hole effects for the Fe K-edge in Fe_3_O_4_ does not significantly impact the results. This approximation is well supported for the O K-edge, but it is important to demonstrate its validity for the Fe K-edge in the context of the present study. The experimental Fe K-edge XANES spectra of Fe_3_O_4_ and the computed ones obtained by the theories considered in our study are compared in Figure 6a. Before getting into the specifics, it is important to note that our current experimental spectrum agrees quite well with previously published data.^85,86^ Interestingly, the simulated and experimental spectra have similar shapes. Moreover, the approximate DFT theory produces an acceptable spectrum. However, there are notable differences in the pre-edge region (0–5 eV) between the simulated and experimental spectra. Specifically, all simulated spectra show a shift toward higher energies in this region compared to the experimental spectrum. Approximate DFT values display a single broad peak in the pre-edge region. On the contrary, implementing Hubbard correction at the DFT + *U* level results in a narrower double peak in this region for both NAO and the OAO projectors. Notably, DFT + *U* + *V* theory presents substantial alterations in the pre-edge compared to DFT + *U*. First, the pre-edge peak is simulated as a single peak for both NAO and OAO projectors. However, when employing the NAO projector to simulate the pre-edge peak, it is shown to be broader compared with that of the OAO projector. Previous studies suggest that the quadrupole-type electronic transitions between 1s and 3d orbitals and the dipole transition between 1s and 4p hybridized with 3d orbitals are responsible for the K pre-edge features.^80,87,88^ Also, due to the broken inversion symmetry in tetrahedral compounds, some p–d hybridization is possible. This means that the probability of absorption increases because of the dipole-allowed transition from the s to the p component of the hybridized states. As a result, the intensity of the pre-edge peak is higher for tetrahedral compounds than for octahedral compounds. This is the case with magnetite (Fe_3_O_4_) and maghemite (γ-Fe_2_O_3_), where some Fe is arranged in tetrahedral coordination.^80^

We now concentrate on the K-edge of Fe_3_O_4_. During O K-edge X-ray absorption, the system is excited from its initial state to a final state by an X-ray with an energy of 530 eV. Herein, it is important to note that in Fe_3_O_4_, each O atom has tetrahedral coordination bonded to four open-shell Fe atoms.^89^Figure 6b shows the experimental and calculations of the O 1s K-edge XANES of Fe_3_O_4_. In general, there are significant similarities between the simulated and experimental spectra. However, DFT + *U* shows a shift to higher energies, mainly at the main edge. Even though the DFT theory simulation agrees very well with the experimental spectrum, it shows an important difference at the main edge, showing a clear splitting between 0 and 2.5 eV, which is not clear in the experimental spectrum. This splitting is also shown in the DFT + *U* + *V*-OAO spectrum with a broader extension (0–3.5 eV). The better agreement is found by using DFT + *U* + *V*-NAO, which is able to reproduce correctly the experimental pre-edge and edge characteristics.

**Figure 7:**
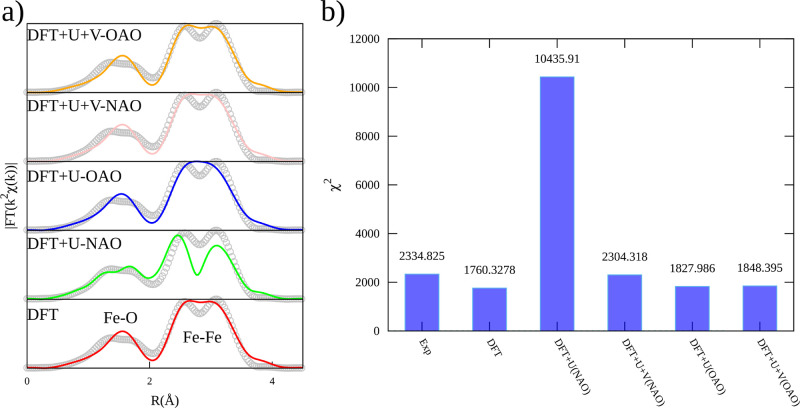
(a) Real part of fits (lines) of the FT signal from *k*^2^-weighted EXAFS signals (dots) and (b) their statistical comparison for the Fe_3_O_4_ bulk crystal by DFT, DFT + *U*, and DFT + *U* + *V* theories.

As mentioned above, EXAFS is a powerful technique used to probe the local atomic structure, providing detailed information about the coordination environment, bond lengths, and disorder around a specific absorbing atom in a sample. The EXAFS oscillations contain valuable structural information encoded in the magnitude, phase, and frequency of the oscillations. By analyzing these oscillations, it is possible to extract the radial distribution function, which describes the distribution of neighboring atoms around the absorbing atom. Then, to ascertain the validity of each model, we compared the obtained structures to experimental EXAFS data at the Fe K-edge. The experimental data and best-fit curves for the EXAFS signal are presented in Figure 7 and Table S2. It is important to note that the same number of parameters was used in the fitting for all theories. For example, the first doublet peak in the range of 1–2 Å (which imitates the Fe_oct_–O and Fe_tet_–O) was modeled by two distances of Fe–O and a single Debye–Waller factor (i.e., one order parameter). On the other hand, the second doublet peak between 2 and 4 Å (which simulates the Fe–Fe bond) has three paths corresponding to three distances and two Debye–Waller factors. For all models, the total amplitude parameter was set to a fixed value of 0.85 (following preliminary adjustment on Fe foil), while the correction to the energy scale was left open to variation. Although Figure 7a shows a close agreement between the experimental and all simulated spectra when analyzing the discrepancy between observed and expected values for the different models (7b), it is evident that DFT + *U*-NAO resulted in the worst experimental data adjustment. However, this model is the only one able to predict a clear splitting of the distribution in the radial range below 2 Å. This can be explained by the fact that the Fe_3_O_4_ structure optimization with the DFT + *U*-NAO theory has a charge disproportionation between Fe^2+^ and Fe^3+^ octahedral sites, which is not captured by the other models. On the contrary, the most accurate curve adjustment to the experimental data was achieved through simulation by approximate DFT and DFT + *U* + *V* (NAO and OAO projectors) methods.

According to the ligand field theory, for octahedral coordination, the 3d atomic orbitals are split into three t_2g_ levels (3d_*xy*_, 3d_*zx*_, and 3d_*zy*_) and two e_g_ levels ( and 3d_*z*^2^_). Then, in order to get a deeper understanding of the XAS signal, we performed the orbital-projected DOS calculations under the different corrected Hubbard approaches. In Figures S2 and S3, we present the orbital projected DOS for the 3d_*xy*_, 3d_*zx*_, 3d_*zy*_, 3d_*z*^2^_5, and  orbitals for both Fe_tet_ and Fe_oct_. However, here we will focus the discussion on Fe_oct_ due to its importance in the electronic and magnetic properties of Fe_3_O_4_. In this regard, our results show that the contributions to the orbital projections to Fe_oct_ are grouped as expected, separated by t_2g_ and e_g_ levels. Around Fermi energy, the DFT predicted that the spin-up valence band and the spin-down conduction band overlapped practically, not presenting any discontinuity, as described previously.^75^ At the DFT + *U* level, the spin down of Fe_oct_ has a large gap, just about t_2g_ states at Fermi energy, extending the discontinuity to the conduction band. On the contrary, at the DFT + *U* + *V* level, the gap is located just below t_2g_ states at Fermi energy. Moreover, the t_2g_ and e_g_ states are slightly separated at the conduction band, which is not true for the DFT + *U* theory. By comparing Figure 6 against Figures S2 and S3, we can conclude that the Fe K pre-edge peak originates mainly from contributions from t_2g_ spin-down orbital states of Fe_oct_ and from e and t_2_ spin-up orbital states of Fe_tet_.

**Figure 8:**
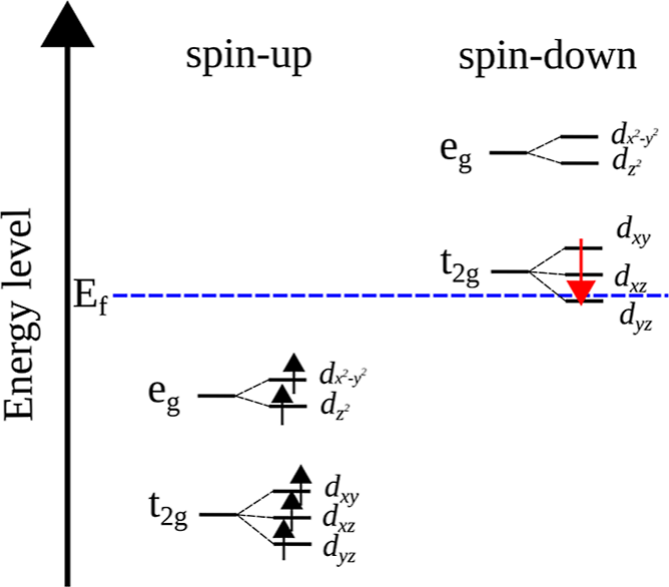
Energy band diagram of the Fe_oct_ 3d orbital in Fe_3_O_4_ bulk crystal calculated by the DFT + *U* + *V* functional.

Taken together, Figure 8 illustrates the energy band diagram of the Fe_oct_ 3d orbital in the Fe_3_O_4_ bulk crystal calculated by the DFT + *U* + *V* theory. All Fe_oct_ spin-up d orbitals are occupied, while most spin-down orbitals remain unoccupied. Nonetheless, the occupation matrix and orbital-projected DOS suggest that an extra electron fractionally occupies the t_2g_ spin-down orbital of Fe_oct_. Due to electron-sharing between Fe_oct_ sites in proximity, there are only fractional occupations of the t_2g_ spin-down orbital. This phenomenon happens because the Fe_oct_ sites share electrons with their neighboring sites and dynamically interconvert through charge hopping. As a result, these levels are not occupied completely, and their occupation matrix eigenvalues are noninteger and range from 0 to 1. Then, above *T*_v_, the Fermi level resides in the Fe_oct_ down-spin t_2g_ band and electric conduction are 100% spin polarized, supporting the half-metallic nature of bulk crystal Fe_3_O_4_.

## 4 Conclusions

In conclusion, using a fully first-principles approach, our study investigated the structural, magnetic, and electronic characteristics of bulk crystal Fe_3_O_4_ above the Verwey temperature. We compared the results obtained from different methods, including DFT, DFT + *U*, DFT + *U* + *V* and HSE (hybrid functional), as well as two types of Hubbard projectors (NAO and OAO). Based on our research, we discovered that the results varied greatly depending on whether *U* corrections alone or both *U* and *V* corrections were employed as well as the type of Hubbard projector. The most optimal agreement was achieved through the utilization of DFT + *U* + *V* along with NAO projectors. The *U* parameter on-site was found to be crucial in describing the confined features of Fe 3d states while the intersite *V* parameter was pertinent in elucidating the robust hybridization between Fe 3d and O 2p states. While the literature often uses the same Hubbard *U* value for Fe_oct_ and Fe_tet_ at the DFT + *U* level, in this case, the HP code detected two distinct values for them through the DFT + *U* and DFT + *U* + *V* levels. Thus, the description of the structural, magnetic, and electronic properties of Fe_3_O_4_ was enhanced by the use of the extended Hubbard functional compared to standard DFT, DFT + *U*, and the HSE functional, without requiring excessive computational resources. Moreover, DFT + *U* + *V* validates the half-metallic character of bulk crystal Fe_3_O_4_ above *T*_v_, since the Fermi level is found in the t_2g_ band with a Fe_oct_ down-spin, indicating that the electric conduction is 100% spin polarized. We believe that DFT + *U* + *V* can play a critical role in improving spectral simulations in materials science, particularly for Fe_3_O_4_. Therefore, our work highlights the significance of considering intersite interactions and the choice of atomic orbital projectors in future theoretical research involving Fe_3_O_4_ not just in its bulk form but also in its various surface and defect configurations. Also, other types of materials such as other transition-metal oxides or materials with mixed valence states could benefit from the use of DFT + *U* + *V* to accurately capture their electronic and magnetic properties and explore their potential applications.

## Supporting Information Available

Calculated net charge of Fe ions, pDOS of the Fe_3_O_4_ bulk crystal, population analysis data, calculated energy difference, bond lengths, interatomic distances, Debye–Waller factors, eigenvalues, occupation matrices, and orbital-projected DOS, (PDF)
