# Supplementary material for: First-Principles Calculations of Magnetite (Fe3O4) above the Verwey Temperature by Using Self-Consistent DFT + U + V
Source: J Chem Theory Comput. 2023 Nov 17;19(23):8610–23. doi: 10.1021/acs.jctc.3c00860 (PMC10720343; doi:10.1021/acs.jctc.3c00860)
Supplement: Supplementary file 1 — ct3c00860_si_001.pdf [file ct3c00860_si_001.pdf]

Supporting Information
First-Principles Calculations of Magnetite (Fe_3O_4) above the Verwey Temperature by Using Self-Consistent DFT + U + V

Nelson Naveas,[*,†,‡,¶] Ruth Pulido,[¶,§] Carlo Marini,[‖] Pierluigi Gargiani,[‖] Jacobo Hernandez-Montelongo,[⊥] Ivan Brito,[§] and Miguel Manso-Silván[†,¶,#]

†*Departamento de Física Aplicada, Universidad Autónoma de Madrid, 28049 Madrid, Spain.*

‡*Departamento de Ingeniería Química y Procesos de Minerales, Universidad de Antofagasta, Avenida Angamos 601, 1270300 Antofagasta, Chile.*

¶*Instituto Universitario de Ciencia de Materiales "Nicolás Cabrera" (INC), Universidad Autónoma de Madrid, Campus de Cantoblanco, 28049 Madrid, Spain*

§*Departamento de Química, Universidad de Antofagasta, Avenida Angamos 601, 1270300 Antofagasta, Chile.*

‖*CELLS–ALBA Synchrotron, 08290 Cerdanyola del Valles, Spain.*

⊥*Departamento de Ciencias Matemáticas y Físicas, UC Temuco, 4813302 Temuco, Chile.*

#*Centro de Microanálisis de Materiales, Universidad Autónoma de Madrid, Campus de Cantoblanco, 28049 Madrid, Spain.*

E-mail: nelson.naveas@estudiante.uam.es

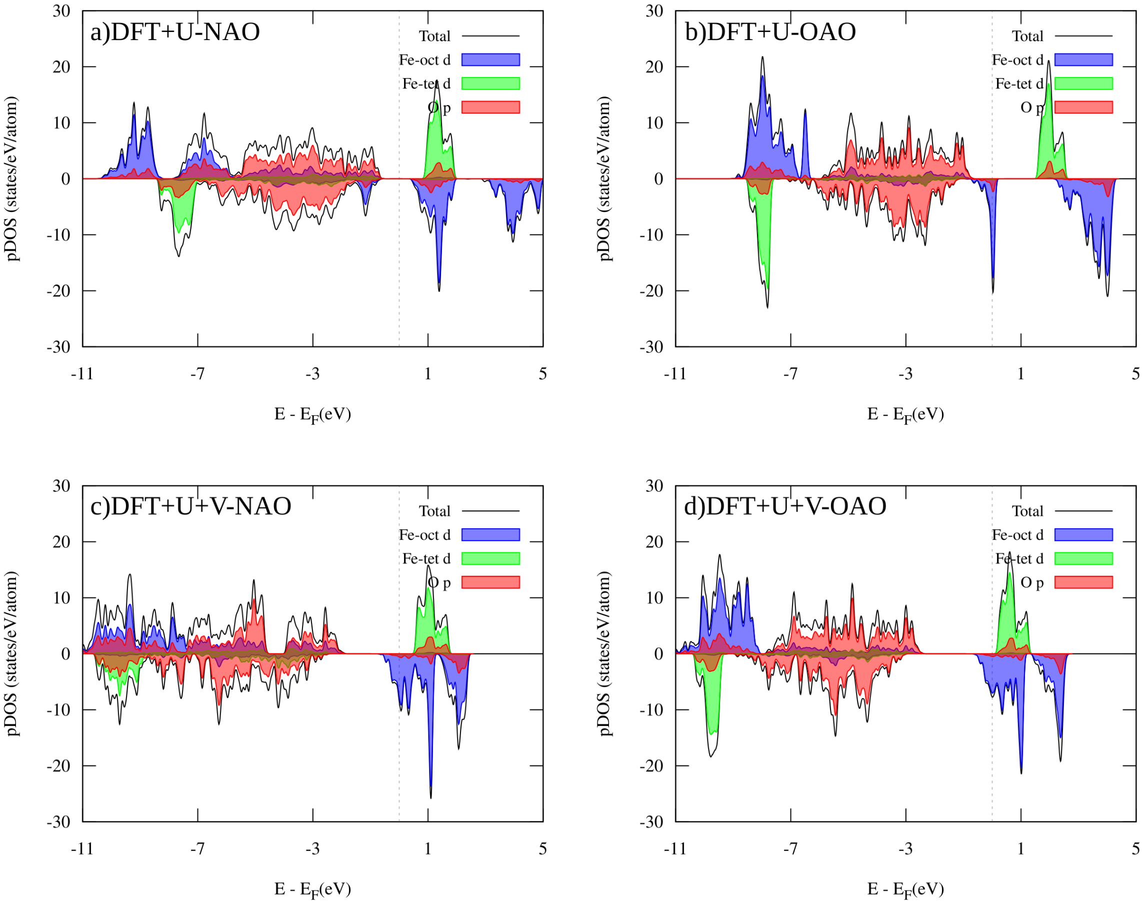

Figure S1: Partial Density of States (pDOS) of the Fe_3O_4 bulk crystal calculated by DFT + U (a and b for NAO and OAO, respectively) and DFT + U + V (c and d for NAO and OAO, respectively) applying no-symmetry constriction.

Table S1: Calculated net charge of Fe ions at tetrahedral and octahedral sites in the Fe_3O_4 primitive cell structure derived from different theories used in the present study.

**Model**	$\mathbf{Fe_{oct}}$	$\mathbf{Fe_{tet}}$
DFT	3.489	3.481
DFT+U-NAO	3.438/3.621	3.654/3.681
DFT+U-OAO	3.575	3.746
DFT+U+V-NAO	3.479	3.578
DFT+U+V-OAO	3.565	3.715

Table S2: Population analysis data for the 3d shell of Fe atoms in the unit cell of Fe_3O_4 derived from different theories used in the present study.

**Theory**	**Atom**	**Spin**	**Ocuppation numbers (d orbitals)**					**OS**
DFT	Fe_{oct}	up	**0.957**	**0.957**	**0.994**	**0.994**	**0.994**	+3
		down	0.298	0.317	0.317	0.368	0.368	
	Fe_{tet}	up	0.335	0.335	0.359	0.359	0.359	+3
		down	**0.957**	**0.957**	**0.957**	**0.989**	**0.989**	
DFT + U-NAO	Fe_{oct1}	up	**0.992**	**0.992**	**0.998**	**0.999**	**0.999**	+3
		down	0.076	0.082	0.243	0.336	0.514	
	Fe_{tet1}	up	0.186	0.186	0.309	0.309	0.347	+3
		down	**0.991**	**0.992**	**0.992**	**0.997**	**0.997**	
	Fe_{oct2}	up	**0.987**	**0.987**	**0.994**	**0.998**	**0.998**	+2
		down	0.053	0.053	0.184	0.184	**0.987**	
	Fe_{tet2}	up	0.186	0.194	0.305	0.305	0.343	+3
		down	**0.991**	**0.991**	**0.992**	**0.997**	**0.997**	
DFT + U-OAO	Fe_{oct}	up	**0.990**	**0.990**	**0.997**	**0.998**	**0.998**	+3
		down	0.043	0.043	0.164	0.164	0.564	
	Fe_{tet}	up	0.170	0.170	0.318	0.318	0.318	+3
		down	**0.991**	**0.991**	**0.991**	**0.997**	**0.997**	
DFT + U + V-NAO	Fe_{oct}	up	**0.990**	**0.990**	**0.998**	**0.998**	**0.998**	+3
		down	0.234	0.234	0.300	0.380	0.380	
	Fe_{tet}	up	0.254	0.254	0.347	0.347	0.347	+3
		down	**0.988**	**0.988**	**0.988**	**0.996**	**0.996**	
DFT + U + V-OAO	Fe_{oct}	up	**0.989**	**0.989**	**0.997**	**0.998**	**0.998**	+3
		down	0.156	0.156	0.217	0.260	0.260	
	Fe_{tet}	up	0.138	0.138	0.222	0.222	0.222	+3
		down	**0.987**	**0.987**	**0.987**	**0.997**	**0.997**	

Table S3: Calculated energy difference per formula unit of Fe_3O_4 between asymmetric and symmetric ground states ($\Delta E = E_{Asym} - E_{Sym}$) derived from the optimized structures obtained by the different theories used in the present study. ΔE_{+UNAO} corresponds to the energy difference by calculations with DFT + U-OAO, DFT + U + V-NAO, and DFT + U + V-OAO theories starting from the symmetry-broken DFT + U-NAO and the high-symmetry structures obtained by the same theories, to assess whether the charge order would arise spontaneously.

**Model**	ΔE (eV)	ΔE_{+UNAO} (eV)
DFT	0.00	0.20
DFT+U-NAO	-0.47	—
DFT+U-OAO	0.00	1.32
DFT+U+V-NAO	0.00	0.09
DFT+U+V-OAO	0.00	0.02

Table S4: Bond lengths for Fe-O and interatomic distances for Fe-Fe, along with the Debye-Waller factors for the first and second shell in Fe_3O_4, according to the different theories used in this study.

	Fe-O				Fe-Fe		
**Model**	R (Å)			**Sigma**	R (Å)		**Sigma**
DFT	1.945	2.049	–	0.011	2.992	3.472	0.01
DFT+U-NAO	1.863	2.064	1.957	0.007	2.928	3.482	0.009
DFT+U-OAO	1.941	2.042	–	0.012	2.996	3.464	0.011
DFT+U+V-NAO	1.945	2.047	–	0.011	2.993	3.470	0.010
DFT+U+V-OAO	1.956	2.047	–	0.011	2.991	3.475	0.010

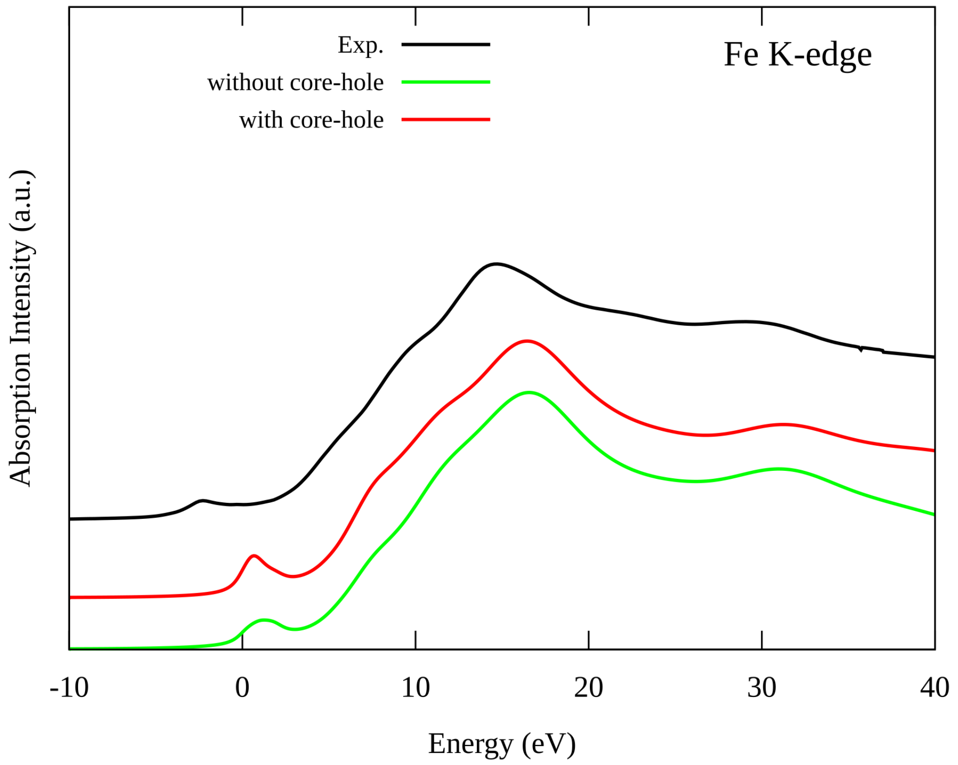

Figure S2: Simulation of Fe_3O_4 bulk Fe K-edge XANES via DFT theory using a pseudopotential approach with and without core-hole effects and their comparison against the experimental spectra.

In our simulations, we computed Fe and O K-edge XANES spectra without taking the core-hole in the final state into account. To establish the accuracy of this method for Fe K-edge, we performed a comparison between simulations that include and exclude core-hole effects of Fe K-edge XAS spectra. For this purpose, the XAS spectra were calculated through the XSPECTRA package[1–3] using a supercell approach that includes a core-hole in the pseudopotential of the absorbing atom. For the supercell, we examined dimensions of $2 \times 2 \times 2$, comprising 112 atoms. Figure S2 presents a comparison of the theoretical results for the Fe K-edge of Fe_3O_4 bulk crystal, with and without the core-hole. The spectra indicate that the presence of the core-hole does not significantly modify the results. In fact, the intensity of the pre-edge peak is more accurately reproduced without the core-hole.

Table S5: Eigenvalues and occupation matrices of the Fe_{oct}-3d orbitals in Fe_3O_4 using the different non-orthogonalized (NAO) Hubbard corrections applied in this study.

**DFT**					**DFT+U-NAO**					**DFT+U+V-NAO**				
**Fe-oct**														
Spin-up eigenvalues:					Spin-up eigenvalues:					Spin-up eigenvalues:				
0.957	0.957	0.994	0.994	0.994	0.991	0.991	0.998	0.999	0.999	0.990	0.990	0.998	0.998	0.998
occupation matrix ns (before diag.):					occupation matrix ns (before diag.):					occupation matrix ns (before diag.):				
0.957	0.000	0.000	0.000	0.000	0.991	0.000	0.000	0.000	0.000	0.990	0.000	0.000	0.000	0.000
0.000	0.994	0.000	0.000	0.000	0.000	0.998	0.000	0.000	0.000	0.000	0.998	0.000	0.000	0.000
0.000	0.000	0.994	0.000	0.000	0.000	0.000	0.998	0.000	0.000	0.000	0.000	0.998	0.000	0.000
0.000	0.000	0.000	0.957	0.000	0.000	0.000	0.000	0.991	0.000	0.000	0.000	0.000	0.990	0.000
0.000	0.000	0.000	0.000	0.994	0.000	0.000	0.000	0.000	0.998	0.000	0.000	0.000	0.000	0.998
Spin-down eigenvalues:					Spin-down eigenvalues:					Spin-down eigenvalues:				
0.298	0.317	0.317	0.368	0.368	0.070	0.070	0.273	0.273	0.615	0.234	0.234	0.300	0.378	0.378
occupation matrix ns (before diag.):					occupation matrix ns (before diag.):					occupation matrix ns (before diag.):				
0.358	-0.009	-0.009	0.000	0.017	0.273	-0.001	-0.001	0.000	0.001	0.346	-0.024	-0.024	0.000	0.049
-0.009	0.318	-0.010	-0.015	-0.010	-0.001	0.252	0.182	-0.001	0.182	-0.024	0.277	0.011	-0.042	0.011
-0.009	-0.010	0.318	0.015	-0.010	-0.001	0.182	0.252	0.001	0.182	-0.024	0.011	0.277	0.042	0.011
0.000	-0.015	0.015	0.358	0.000	0.000	-0.001	0.001	0.273	0.000	0.000	-0.042	0.042	0.346	0.000
0.017	-0.010	-0.010	0.000	0.318	0.001	0.182	0.182	0.000	0.252	0.049	0.011	0.011	0.000	0.277

Table S6: Eigenvalues and occupation matrices of the Fe_{tet}-3d orbitals in Fe_3O_4 using the different non-orthogonalized (NAO) Hubbard corrections applied in this study.

**DFT**					**DFT+U-NAO**					**DFT+U+V-NAO**				
**Fe-tet**														
Spin-up					Spin-up					Spin-up				
eigenvalues:					eigenvalues:					eigenvalues:				
0.335	0.335	0.359	0.359	0.359	0.191	0.191	0.321	0.321	0.321	0.253	0.253	0.346	0.346	0.346
occupation matrix ns (before diag.):					occupation matrix ns (before diag.):					occupation matrix ns (before diag.):				
0.335	0.000	0.000	0.000	0.000	0.191	0.000	0.000	0.000	0.000	0.253	0.000	0.000	0.000	0.000
0.000	0.359	0.000	0.000	0.000	0.000	0.321	0.000	0.000	0.000	0.000	0.346	0.000	0.000	0.000
0.000	0.000	0.359	0.000	0.000	0.000	0.000	0.321	0.000	0.000	0.000	0.000	0.346	0.000	0.000
0.000	0.000	0.000	0.335	0.000	0.000	0.000	0.000	0.191	0.000	0.000	0.000	0.000	0.253	0.000
0.000	0.000	0.000	0.000	0.359	0.000	0.000	0.000	0.000	0.321	0.000	0.000	0.000	0.000	0.346
eigenvalues:					eigenvalues:					eigenvalues:				
0.957	0.957	0.957	0.989	0.989	0.989	0.989	0.989	0.997	0.997	0.988	0.988	0.988	0.996	0.996
occupation matrix ns (before diag.):					occupation matrix ns (before diag.):					occupation matrix ns (before diag.):				
0.989	0.000	0.000	0.000	0.000	0.997	0.000	0.000	0.000	0.000	0.996	0.000	0.000	0.000	0.000
0.000	0.957	0.000	0.000	0.000	0.000	0.989	0.000	0.000	0.000	0.000	0.988	0.000	0.000	0.000
0.000	0.000	0.957	0.000	0.000	0.000	0.000	0.989	0.000	0.000	0.000	0.000	0.988	0.000	0.000
0.000	0.000	0.000	0.989	0.000	0.000	0.000	0.000	0.997	0.000	0.000	0.000	0.000	0.996	0.000
0.000	0.000	0.000	0.000	0.957	0.000	0.000	0.000	0.000	0.989	0.000	0.000	0.000	0.000	0.988

Table S7: Eigenvalues and occupation matrices of the Fe_{oct}-3d orbitals in Fe_3O_4 using the different orthogonalized (OAO) Hubbard corrections applied in this study.

**DFT**					**DFT+U-OAO**					**DFT+U+V-OAO**				
**Fe-oct**														
Spin-up eigenvalues:					Spin-up eigenvalues:					Spin-up eigenvalues:				
0.957	0.957	0.994	0.994	0.994	0.992	0.992	0.998	0.999	0.999	0.989	0.989	0.997	0.998	0.998
occupation matrix ns (before diag.):					occupation matrix ns (before diag.):					occupation matrix ns (before diag.):				
0.957	0.000	0.000	0.000	0.000	0.992	0.000	0.000	0.000	0.000	0.989	0.000	0.000	0.000	0.001
0.000	0.994	0.000	0.000	0.000	0.000	0.998	0.000	0.000	0.000	0.000	0.998	0.000	-0.001	0.000
0.000	0.000	0.994	0.000	0.000	0.000	0.000	0.998	0.000	0.000	0.000	0.000	0.998	0.001	0.000
0.000	0.000	0.000	0.957	0.000	0.000	0.000	0.000	0.992	0.000	0.000	-0.001	0.001	0.989	0.000
0.000	0.000	0.000	0.000	0.994	0.000	0.000	0.000	0.000	0.998	0.001	0.000	0.000	0.000	0.998
Spin-down eigenvalues:					Spin-down eigenvalues:					Spin-down eigenvalues:				
0.298	0.317	0.317	0.368	0.368	0.066	0.066	0.267	0.267	0.618	0.156	0.156	0.217	0.259	0.259
occupation matrix ns (before diag.):					occupation matrix ns (before diag.):					occupation matrix ns (before diag.):				
0.358	-0.009	-0.009	0.000	0.017	0.267	-0.001	-0.001	0.000	0.001	0.206	-0.021	-0.021	0.000	0.042
-0.009	0.318	-0.010	-0.015	-0.010	-0.001	0.250	0.184	-0.001	0.184	-0.021	0.212	0.003	-0.036	0.003
-0.009	-0.010	0.318	0.015	-0.010	-0.001	0.184	0.250	0.001	0.184	-0.021	0.003	0.212	0.036	0.003
0.000	-0.015	0.015	0.358	0.000	0.000	-0.001	0.001	0.267	0.000	0.000	-0.036	0.036	0.206	0.000
0.017	-0.010	-0.010	0.000	0.318	0.001	0.184	0.184	0.000	0.250	0.042	0.003	0.003	0.000	0.212

Table S8: Eigenvalues and occupation matrices of the Fe_{tet}-3d orbitals in Fe_3O_4 using the different non-orthogonalized (NAO) Hubbard corrections applied in this study.

**DFT**					**DFT+U-OAO**					**DFT+U+V-OAO**				
**Fe-tet**														
Spin-up					Spin-up					Spin-up				
eigenvalues:					eigenvalues:					eigenvalues:				
0.335	0.335	0.359	0.359	0.359	0.170	0.170	0.318	0.318	0.318	0.138	0.138	0.221	0.221	0.221
occupation matrix ns (before diag.):					occupation matrix ns (before diag.):					occupation matrix ns (before diag.):				
0.335	0.000	0.000	0.000	0.000	0.170	0.000	0.000	0.000	0.000	0.138	0.000	0.000	0.000	0.000
0.000	0.359	0.000	0.000	0.000	0.000	0.318	0.000	0.000	0.000	0.000	0.221	0.000	0.000	0.000
0.000	0.000	0.359	0.000	0.000	0.000	0.000	0.318	0.000	0.000	0.000	0.000	0.221	0.000	0.000
0.000	0.000	0.000	0.335	0.000	0.000	0.000	0.000	0.170	0.000	0.000	0.000	0.000	0.138	0.000
0.000	0.000	0.000	0.000	0.359	0.000	0.000	0.000	0.000	0.318	0.000	0.000	0.000	0.000	0.221
eigenvalues:					eigenvalues:					eigenvalues:				
0.957	0.957	0.957	0.989	0.989	0.991	0.991	0.991	0.997	0.997	0.987	0.987	0.987	0.997	0.997
occupation matrix ns (before diag.):					occupation matrix ns (before diag.):					occupation matrix ns (before diag.):				
0.989	0.000	0.000	0.000	0.000	0.997	0.000	0.000	0.000	0.000	0.997	0.000	0.000	0.000	0.000
0.000	0.957	0.000	0.000	0.000	0.000	0.991	0.000	0.000	0.000	0.000	0.987	0.000	0.000	0.000
0.000	0.000	0.957	0.000	0.000	0.000	0.000	0.991	0.000	0.000	0.000	0.000	0.987	0.000	0.000
0.000	0.000	0.000	0.989	0.000	0.000	0.000	0.000	0.997	0.000	0.000	0.000	0.000	0.997	0.000
0.000	0.000	0.000	0.000	0.957	0.000	0.000	0.000	0.000	0.991	0.000	0.000	0.000	0.000	0.987

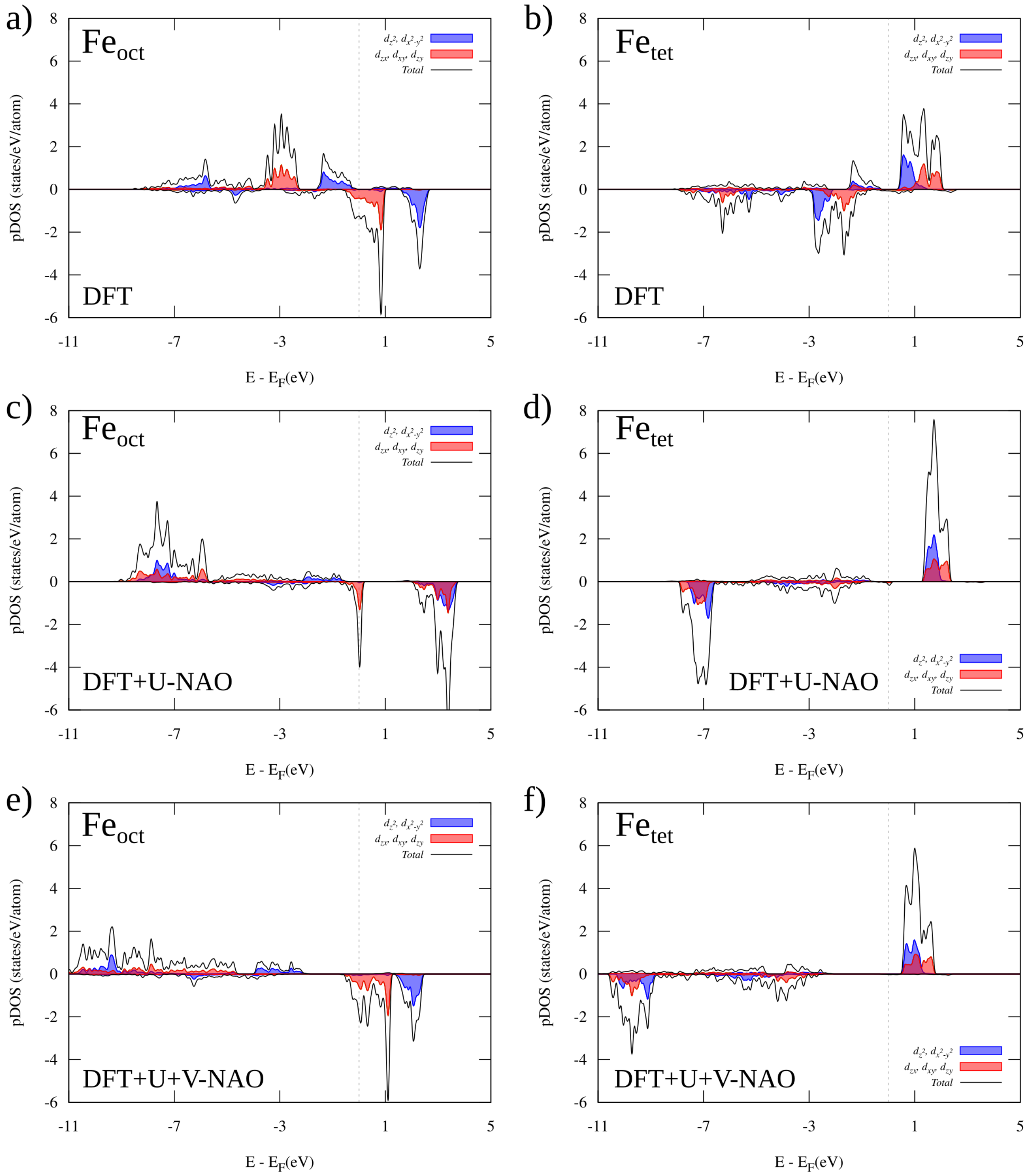

Figure S3: Orbital-projected Density of States (DOS) of the Fe 3d states of Fe_3O_4 bulk crystal calculated by DFT and Hubbard approaches using NAO projectors.

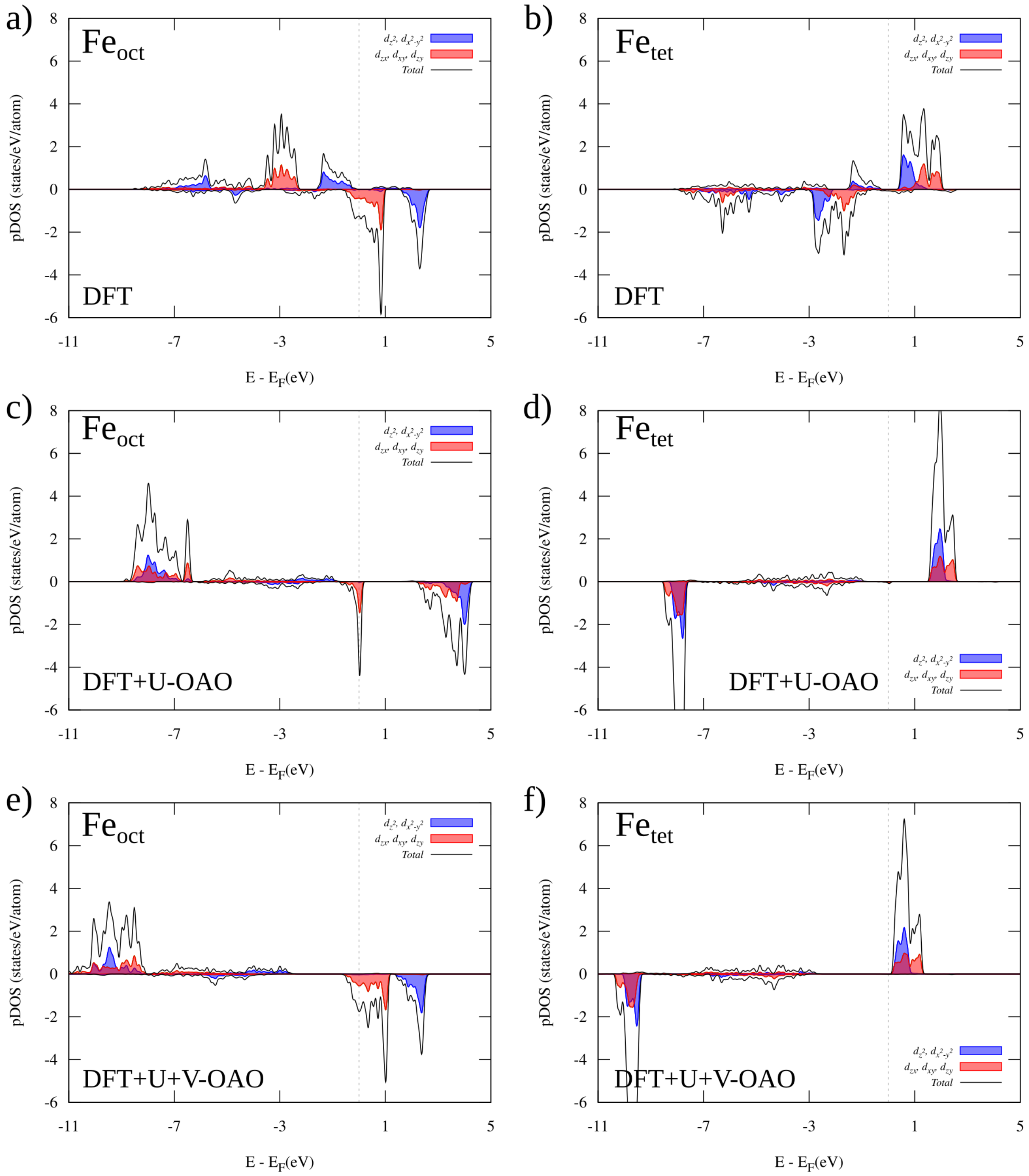

Figure S4: Orbital-projected Density of States (DOS) of the Fe 3d states of Fe_3O_4 bulk crystal calculated by DFT and Hubbard approaches using OAO projectors.

References

(1) Taillefumier, M.; Cabaret, D.; Flank, A.-M.; Mauri, F. X-ray absorption near-edge structure calculations with the pseudopotentials: Application to the K edge in diamond and

α-quartz. *Phys. Rev. B* **2002**, *66*, 195107.

(2) Gougoussis, C.; Calandra, M.; Seitsonen, A.; Brouder, C.; Shukla, A.; Mauri, F. Intrinsic charge transfer gap in NiO from Ni K-edge x-ray absorption spectroscopy. *Phys. Rev. B* **2009**, *79*, 045118.

(3) Timrov, I.; Agrawal, P.; Zhang, X.; Erat, S.; Liu, R.; Braun, A.; Cococcioni, M.; Calandra, M.; Marzari, N.; Passerone, D. Electronic structure of pristine and Ni-substituted $LaFeO_3$ from near edge x-ray absorption fine structure experiments and first-principles simulations. *Phys. Rev. Res.* **2020**, *2*, 033265.
